# Supplementary material for: Physical Activity and Its Diurnal Fluctuations Vary by Non-Motor Symptoms in Patients with Parkinson’s Disease: An Exploratory Study
Source: Healthcare (Basel). 2022 Apr 18;10(4):749. doi: 10.3390/healthcare10040749 (PMC9029803; doi:10.3390/healthcare10040749)
Supplement: Supplementary file 1 [file healthcare-10-00749-s001.zip › healthcare-1586338-supplementary.pdf]

Supplementary

Table S1. Comparison of step counts with and non NMS by ANCOVA [step counts: continuous scale].

	With NMS		Without NMS			
	Mean	SE	Mean	SE	f-value	*p*-value
Cognitive impairment	-	-	-	-	-	-
Hallucinations and psychosis	-	-	-	-	-	-
Depressed mood						
Anxious mood	-	-	-	-	-	-
Apathy	-	-	-	-	-	-
Features of dopamine dysregulation syndrome	-	-	-	-	-	-
Sleep problems	4359.34	(3237.50–5481.18)	4670.03	(3796.58–5543.48)	0.19	0.66
Daytime sleepiness	4363.43	(3117.11–5609.75)	4657.06	(3761.12–5553.00)	0.14	0.71
Pain and other sensations	3600.18	(2670.11–4530.25)	5463.72	(4555.30–6372.15)	7.92	<0.01
Urinary problems	4707.82	(3436.60–5679.04)	4482.58	(3641.41–5323.76)	0.09	0.77
Constipation problems	4501.12	(3566.28–5436.02)	4629.95	(3456.57–5801.35)	0.03	0.87
Light-headedness on standing	-	-	-	-	-	-
Fatigue	3225.07	(2161.08–4289.06)	5358.69	(4546.59–6170.80)	9.62	<0.01

95% CI, 95% confidence interval of mean; NMS, non-motor symptoms. The covariate controlled for age, gender, and the mHY stage.

Table S2. Association between MVPA and NMS by logistic regression analysis [dependent variable: insufficient MVPA (< 150 min/week)].

	Crude model			Adjusted model		
	OR	95% CI	p-value	OR	95% CI	*p*-value
Cognitive impairment	-	-	-	-	-	-
Hallucinations and psychosis	-	-	-	-	-	-
Depressed mood	1.65	(0.32–8.39)	0.55	0.69	(0.83–5.79)	0.74
Anxious mood	-	-	-	-	-	-
Apathy	-	-	-	-	-	-
Features of dopamine dysregulation syndrome	-	-	-	-	-	-
Sleep problems	1.03	(0.31–3.43)	0.97	1.17	(0.24–5.75)	0.84
Daytime sleepiness	6.67	(1.68–26.45)	<0.01	3.61	(0.67–19.37)	0.13
Pain and other sensations	4.00	(1.16–13.86)	0.03	5.93	(0.99–35.69)	0.05
Urinary problems	1.85	(0.52–6.62)	0.35	8.24	(0.24–8.24)	0.70
Constipation problems	3.78	(1.05–13.68)	0.04	10.17	(1.26–82.31)	0.03
Light-headedness on standing	-	-	-	-	-	-
Fatigue	5.07	(1.37–18.79)	0.02	10.13	(1.39–73.82)	0.02

OR, odds ratio; 95% CI, 95% confidence interval of odds ratio; MVPA, moderate-to-vigorous physical activity; NMS, non-motor symptoms. The adjusted model statistically controlled for age, gender, and the mHY stage.

Table S3. Association between LPA and non-motor symptoms by linear regression analysis (dependent variable: log-transformed LPA).

	Crude model			Adjusted model		
	CE	95% CI	*p*-value	CE	95% CI	*p*-value
Cognitive impairment	-75.04	(-176.86, 26.77)	0.14	-30.36	(-108.96, 48.25)	0.44
Hallucinations and psychosis	-7.85	(-150.10, 134.40)	0.91	37.58	(-68.60, 143.76)	0.48
Depressed mood	49.82	(-46.90, 146.53)	0.16	48.79	(-24.83, 122.40)	0.19
Anxious mood	-8.00	(-128.35, 112.35)	0.89	30.96	(-60.07, 121.99)	0.50
Apathy	80.68	(-41.53, 202.89)	0.19	41.76	(-52.65, 136.17)	0.38
Features of dopamine dysregulation syndrome	-16.00	(-256.71, 224.71)	0.89	61.92	(-120.13, 243.97)	0.50
Sleep problems	6.42	(-66.76, 79.59)	0.86	-0.83	(-54.80, 53.14)	0.98
Daytime sleepiness	64.48	(-6.96, 135.92)	0.08	31.51	(-28.70, 91.73)	0.30
Pain and other sensations	-11.67	(-82.58, 59.23)	0.74	-42.05	(-95.78, 11.68)	0.12
Urinary problems	-31.44	(-107.48, 44.61)	0.41	-28.06	(-86.07, 29.95)	0.33
Constipation problems	-3.57	(-75.99, 68.87)	0.92	15.03	(-44.47, 74.53)	0.61
Light-headedness on standing	35.81	(-136.04, 207.67)	0.68	67.76	(-57.89, 193.41)	0.28
Fatigue	75.44	(6.01, 114.86)	0.03	39.80	(-17.35, 96.96)	0.17

CE, coefficient estimate; 95% CI, 95% confidence interval of coefficient estimate; LPA, light physical activity; NMS, non-motor symptoms. The adjusted model statistically controlled for age, gender, and the mHY stage.

Table S4. Association between SB and NMS by linear regression analysis (dependent variable: log-transformed SB).

	Crude model			Adjusted model		
	CE	95% CI	*p*-value	CE	95% CI	*p*-value
Cognitive impairment	-0.97	(-72.97, 71.03)	0.98	1.89	(-74.85, 78.63)	0.96
Hallucinations and psychosis	-8.25	(-106.34, 89.84)	0.87	-9.59	(-113.07, 93.91)	0.85
Depressed mood	-63.72	(-128.35, 0.90)	0.05	-69.43	(-138.88, 0.02)	0.05
Anxious mood	-10.13	(-93.10, 72.83)	0.81	-9.42	(-98.09, 79.24)	0.83
Apathy	2.43	(-83.574, 88.432)	0.96	3.77	(-88.61, 96.15)	0.94
Features of dopamine dysregulation syndrome	-20.27	(-186.19, 145.66)	0.81	-18.85	(-196.18, 158.49)	0.83
Sleep problems	-37.83	(-86.96, 11.29)	0.13	-37.24	(-88.16, 13.69)	0.15
Daytime sleepiness	-14.23	(-65.17, 36.72)	0.58	-14.75	(-73.72, 44.22)	0.62
Pain and other sensations	51.72	(5.41, 98.03)	0.03	53.56	(2.70, 104.42)	0.04
Urinary problems	-37.79	(-89.36, 13.89)	0.15	-42.36	(-97.60, 12.88)	0.13
Constipation problems	-34.60	(-83.41, 14.22)	0.16	-49.97	(-105.56, 5.62)	0.08
Light-headedness on standing	-71.07	(-187.82, 45.67)	0.23	-76.37	(-197.48, 44.74)	0.21
Fatigue	-10.91	(-61.28, 39.46)	0.66	-5.26	(-61.98, 51.45)	0.85

CE, coefficient estimate; 95% CI, 95% confidence interval of coefficient estimate; SB, sedentary behavior; NMS, non-motor symptoms. The adjusted model statistically controlled for age, gender, and the mHY stage.
